# Behavioral Observations From a Mountain Lion Nursery in a Recolonizing Great Plains Population

**DOI:** 10.1002/ece3.71687

**Published:** 2025-07-10

**Authors:** Maximilian L. Allen, Colin Croft, Shannon P. Finnegan, Bethany H. Warner

**Affiliations:** ^1^ Illinois Natural History Survey Prairie Research Institute, University of Illinois Champaign Illinois USA; ^2^ Western Nebraska Community College Scottsbluff Nebraska USA; ^3^ Research Department Leopard Ecology and Conservation Gaborone Botswana

**Keywords:** camera trap, den, detection, nursery, *Puma concolor*, vocalization

## Abstract

Natal dens and neonatal behavior are a critical but understudied component of reproductive success in cryptic solitary carnivores. Mountain lion ( *Puma concolor* ) nursery sites tend to be in remote and difficult‐to‐observe areas—as a result, there are few in‐depth behavioral observations of neonatal mountain lions. In this study, we present detailed opportunistic observations from a week of continuous monitoring of a mountain lion nursery located in the Gilbert‐Baker Wildlife Management Area in northwestern Nebraska. Using a non‐invasive video camera trap, we recorded 403 videos comprising 76 distinct bouts of activity. We confirmed the presence of four kittens during the first day of monitoring; however, subsequent recordings consistently captured three or fewer individuals—with most videos featuring one (50.7%) or two (34.8%) kittens. This variation demonstrates the inherent limitations of camera traps to reliably detect all individuals, potentially biasing abundance estimates that rely on repeated count data. Play (chasing, pouncing, wrestling, and tree climbing) and vocalizations (high‐pitched contact calls by kittens and low growling purrs from the mother) were the most frequent behaviors we observed. We also documented allogrooming and autogrooming, as well as the mother carrying kittens in her mouth. Although no nursing or feeding was directly observed, prey remains found near the nursery on a subsequent visit confirmed maternal foraging and provisioning. Activity patterns were crepuscular overall but differed by mountain lion class and temporal resolution. Specifically, these patterns varied depending on whether we analyzed times from individual videos or the beginning of activity bouts, underscoring the importance of the data resolution used when analyzing camera trap data. Our findings demonstrate the value of camera traps for studying neonatal carnivore behavior and highlight the need for suitable nursery habitats as mountain lions recolonize portions of their former range.

## Graphical abstract

We were able to make unique observations of a mountain lion family from a short period of monitoring a nursery. Playing was the most frequent behavior we documented at the nursery, along with vocalizations, grooming, and other behaviors. Our findings demonstrate the value of camera traps for studing the behavior of young animals, but also highlight the importance of nursery habitats for mountain lions as they recolonize new parts of their range.

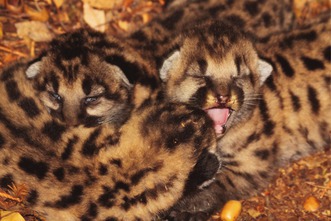

## 1 Introduction

For many mammals, natal dens are a crucial aspect of protection for their neonatal young during their most vulnerable life stage. Neonatal mortality is high in many mammal species due to their greater susceptibility to predation and environmental stressors (Caughley 1966; Promislow and Harvey 1990). This heightened risk and need for protection from predators and elements is compounded by neonatal young having high energetic demands (Yovovich et al. 2020; Oftedal and Glittleman 1989). This often leads to denning behavior being synchronized with periods of high food availability to allow mothers to meet the high energetic demands of their offspring, as well as the high energetic demands associated with lactation and increased hunting (Jansen and Jenks 2012; Smereka et al. 2020; McHuron et al. 2023). The availability, quality, and distribution of suitable denning habitats therefore directly affect the reproductive success of animal populations (Elbroch et al. 2015; Matthews et al. 2019). Recent findings also highlight the importance of maternal experience in shaping both den site selection and reproductive success—with experienced females more likely to choose sites that enhance offspring survival. A greater understanding of natal dens is therefore essential for informing conservation and management strategies, particularly in the context of accelerating habitat loss and climate change.

**FIGURE 1:**
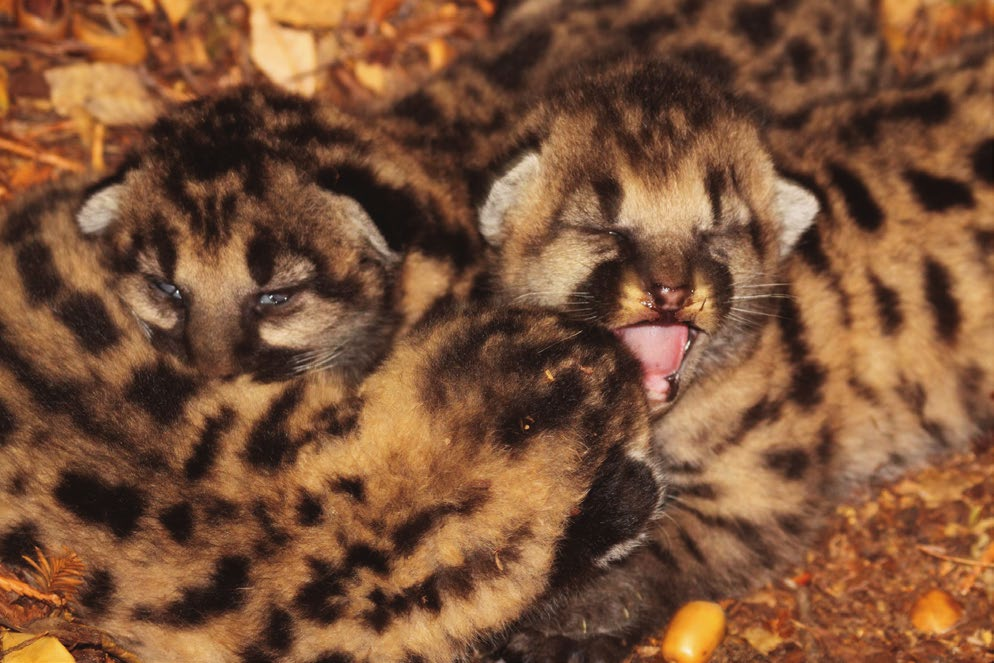
Young mountain lion ( *Puma concolor* ) kittens in a nursery (photo by Max Allen).

Mountain lions ( *Puma concolor* ; Figure 1) are solitary apex carnivores that are integral to many ecosystems as keystone species (Avrin et al. 2023). Male mountain lions regularly scent mark to advertise their status for potential females, and females tend to select for the dominant territorial males in the area to mate with (i.e., older and larger males that scent mark most regularly; Allen et al. 2015). Mating can take place at any time of year, although some populations do exhibit birth pulses that coincide with high prey availability (Lindzey et al. 1994; Jansen and Jenks 2012; Elbroch et al. 2015). Mountain lions give birth to 1–4 kittens (usually 2–3; Lindzey et al. 1994), after a gestation period of around 3 months (i.e., 82–96 days; Currier 1983), and kittens in smaller litters tend to have greater mass (Jansen and Jenks 2012). Mountain lions tend to use nursery sites (typically protected areas like thickets), rather than a den that is dug into the ground (Maehr et al. 1989; Benson et al. 2008; Elbroch et al. 2015). The nursery sites tend to be in forest habitats with little human development (Wilmers et al. 2013; Yovovich et al. 2020), and are typically used for 2–3 months for rearing of neonatal young (Beier et al. 1995; Benson et al. 2008). The home range size of females decreases during denning, as mountain lions become central place foragers (Maehr et al. 1989; Beier et al. 1995; Yovovich et al. 2020), and females tend to increase their foraging time away from nurseries as kittens grow older (Maehr et al. 1989). Kittens stay with their mothers for 12–18 months, with males typically dispersing before females (Jansen and Jenks 2012).

Neonatal kittens tend to have higher mortality than older kittens (Clark et al. 2015), but are difficult to observe over long time periods. As a result, there are few in‐depth behavioral observations of neonatal mountain lions (but see Maehr et al. 1989). Here we report on observations from a one‐week snapshot of monitoring a mountain lion nursery in the Gilbert‐Baker Wildlife Management Area in northwestern Nebraska with a video camera trap. Our objectives were to document the activity and behavior of mountain lions at the nursery, including quantifying patterns of activity and the number of kittens observed, documenting specific behaviors observed, and determining and comparing the diel activity of the mother and kittens.

## 2 Materials and Methods

### 2.1 Study Area

Our observations are from the Gilbert‐Baker Wildlife Management Area, in the sparsely human‐populated Sioux County of Nebraska. This 2537‐acre Wildlife Management Area lies within the larger Pine Ridge ecosystem, which has been designated a Biologically Unique Landscape under Nebraska's Natural Legacy Project (Nebraska Natural Legacy Project 2024). Elevation ranges from around 1341 m to just over 1524 m. Gilbert‐Baker experiences a semi‐arid climate characterized by cold winters and warm summers. Annual precipitation ranges from 355 to 457 mm, most of which occurs from late spring to early summer. Significant temperature fluctuations are common; average January temperatures range from lows near −11°C to highs around 3°C, while July temperatures typically range from lows near 14°C to highs around 31°C. The topography of the Pine Ridge has distinctive rocky outcrops, buttes, steep ridges, and deep and densely vegetated canyons. Like other areas of the Pine Ridge, ponderosa pine *((Pinus ponderosa* ) is the dominant woodland species at Gilbert‐Baker, with rocky mountain juniper ( *Juniperus scopulorum* ) in the subcanopy, along with abundant American plum *Prunus americana* ) and chokecherry ( *Prunus virginiana* ) often forming thick stands in lower‐lying and moister areas. Deciduous species, particularly eastern cottonwood ( *Populus deltoides* ), willow (*Salix* spp.), and boxelder ( *Acer negundo* ) are found in the watered ravines. Riverbank grape ( *Vitis riparia* ) and Virginia creeper ( *Parthenocissus quinquefolia* ) often grow thick and tangled around some of the larger trees. This habitat diversity supports a variety of animal species, including important mountain lion prey such as white‐tailed deer *(Odocoileus virginianus* ), mule deer ( *Odocoileus hemionus* ), and elk ( *Cervus canadensis* ). Other notable species include reintroduced bighorn sheep ( *Ovis canadensis* ), bobcat ( *Lynx rufus* ), coyote ( *Canis latrans* ), and wild turkey ( *Meleagris gallopavo* ).

### 2.2 Field Methods

We deployed a video camera trap (Browning Recon Force Elite BTC‐7E‐HP5, Birmingham, AL, USA) during multiple time periods in the general location of the nursery for approximately 2 years (from January 2023 to January 2025). The camera trap was placed in order to document mountain lion activity in the area, and we placed it on a trail that was periodically used by mountain lions. We programmed the camera to be active 24 h a day and triggered by motion within the viewshed. The first concentrated activity of mountain lions using the nursery was on November 1, 2024, about 10 m from the camera trap, and the camera trap remained in this location capturing footage until November 7, 2024, when the 128 GB memory card was full.

### 2.3 Statistical Analyses

We used R version 4.4.1 (R Core Team 2024) for our statistical analyses. We used two complementary metrics to quantify mountain lion visitation: the number of visitation bouts (defined as discrete periods of activity separated by inactivity) and the total number of video recordings. We categorized mountain lion behaviors we observed in the videos into five categories (Table 1), and classified vocalizations of the mother and the offspring by comparing them with previous research (Allen et al. 2016).

**TABLE 1:** An ethogram describing mountain lion behaviors that could potentially be observed at nursery sites through direct observations or camera trap footage, categorized by behavioral function and described for use in field‐based behavioral studies.

Behavior	Definition
Playing	Where an animal exhibits non‐aggressive and potentially spontaneous activity that includes chasing, pouncing, wrestling, and tree climbing (Videos 1 and 2)
Nursing	Where a kitten attaches its mouth to a nipple and suckles from the mother
Eating	Where an animal ingests solid food into the mouth, chewing, and swallowing
Vocalizations	Where a sound is produced by the mother (typically a low growling purr; Video 1) or kitten (typically a high‐pitched chirp as a contact call; Video 2)
Grooming	Where an animal cleans and cares for fur through licking with tongue (Video 3), either as allogrooming (grooming of a conspecific) or autogrooming (grooming of oneself)

**VIDEO 1:** Two mountain lion kittens playing (climbing trees and chasing), as the mother watches and makes a vocalization. Video content can be viewed at https://onlinelibrary.wiley.com/doi/10.1002/ece3.71687 Video content can be viewed at https://onlinelibrary.wiley.com/doi/10.1002/ece3.71687.

**VIDEO 2:** Vocalizations by mother mountain lion to get the attention of a kitten, who then approaches and plays with the mother's tail. Video content can be viewed at https://onlinelibrary.wiley.com/doi/10.1002/ece3.71687 Video content can be viewed at https://onlinelibrary.wiley.com/doi/10.1002/ece3.71687.

To assess the diel activity of mountain lions at the nursery, we split the mountain lions into three classes (only the mother, only kittens, and the mother and kittens together) and performed analyses of their diel activity using two data sets: the time each visitation bout began and the time each video began. We first transformed all time data to solar time using the *solartime* package (Wutzler 2021) and then grouped visits by class (mother, kittens, mother and kittens). We then created plots of diel activity for each mountain lion class for each data source using the *overlap* package (Meredith and Ridout 2014).

## 3 Results

**FIGURE 2:**
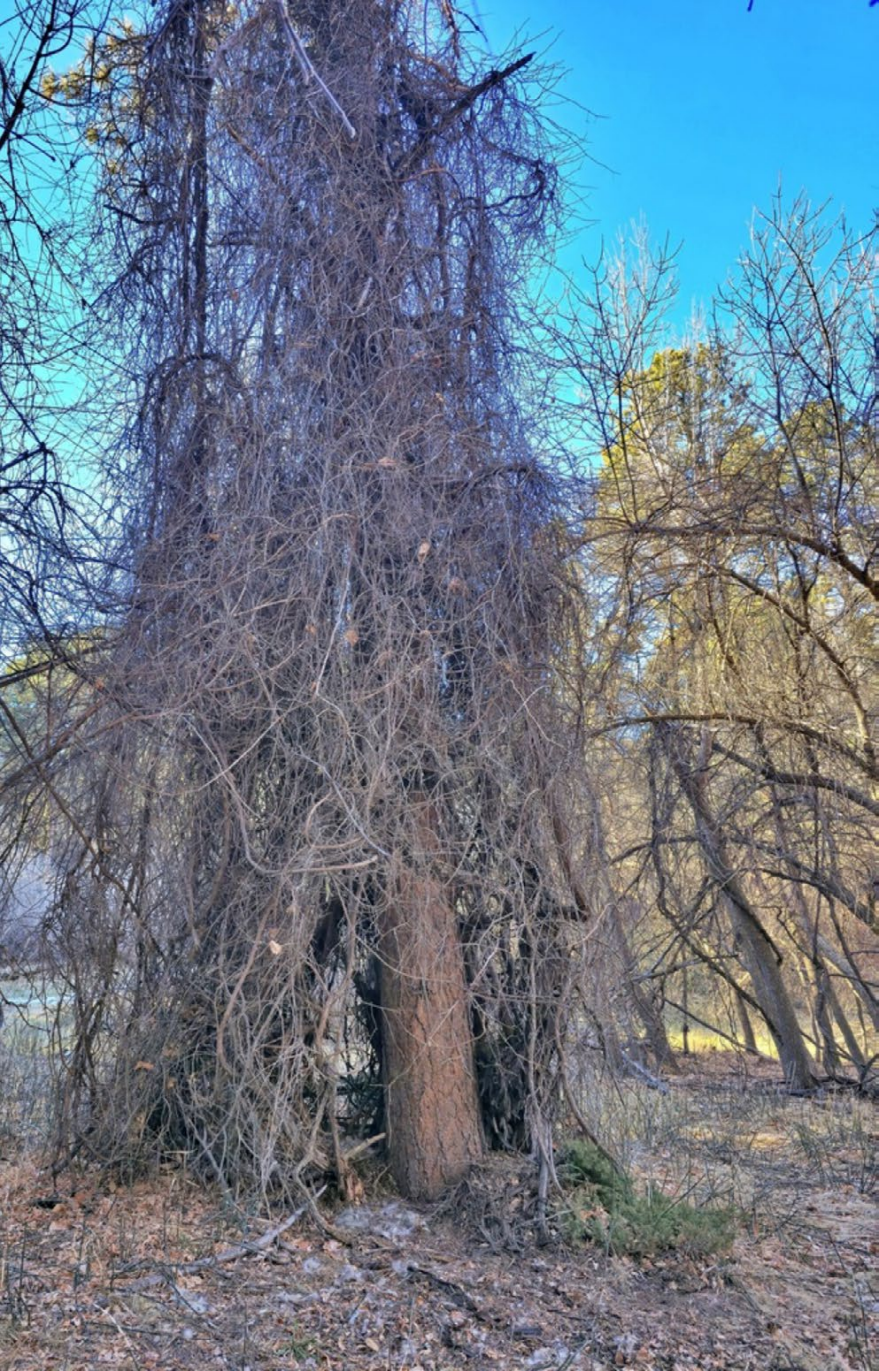
The mountain lion nursery site in the Gilbert‐Baker Wildlife Management Area, Nebraska. The nursery was located in the hollow portion of the river grape vines on the ponderosa pine tree (Photo by Colin Croft).

The mountain lion nursery observed in this study was located in the hollow area of a dense mat of River grape that grew around the lower limbs of a ponderosa pine (Figure 2). The camera trap was not facing directly into the nursery, leading to documentation of activity around the general area of the nursery. Over the seven‐day observation period when the nursery and video camera trap were active (November 1–November 7, 2024), we recorded a total of 403 videos of 76 distinct bouts of activity. Of these activity bouts, 26 featured only the adult female (127 videos), 9 included only kittens (155 videos), and 41 bouts documented the presence of both the mother and kittens (121 videos).

**FIGURE 3:**
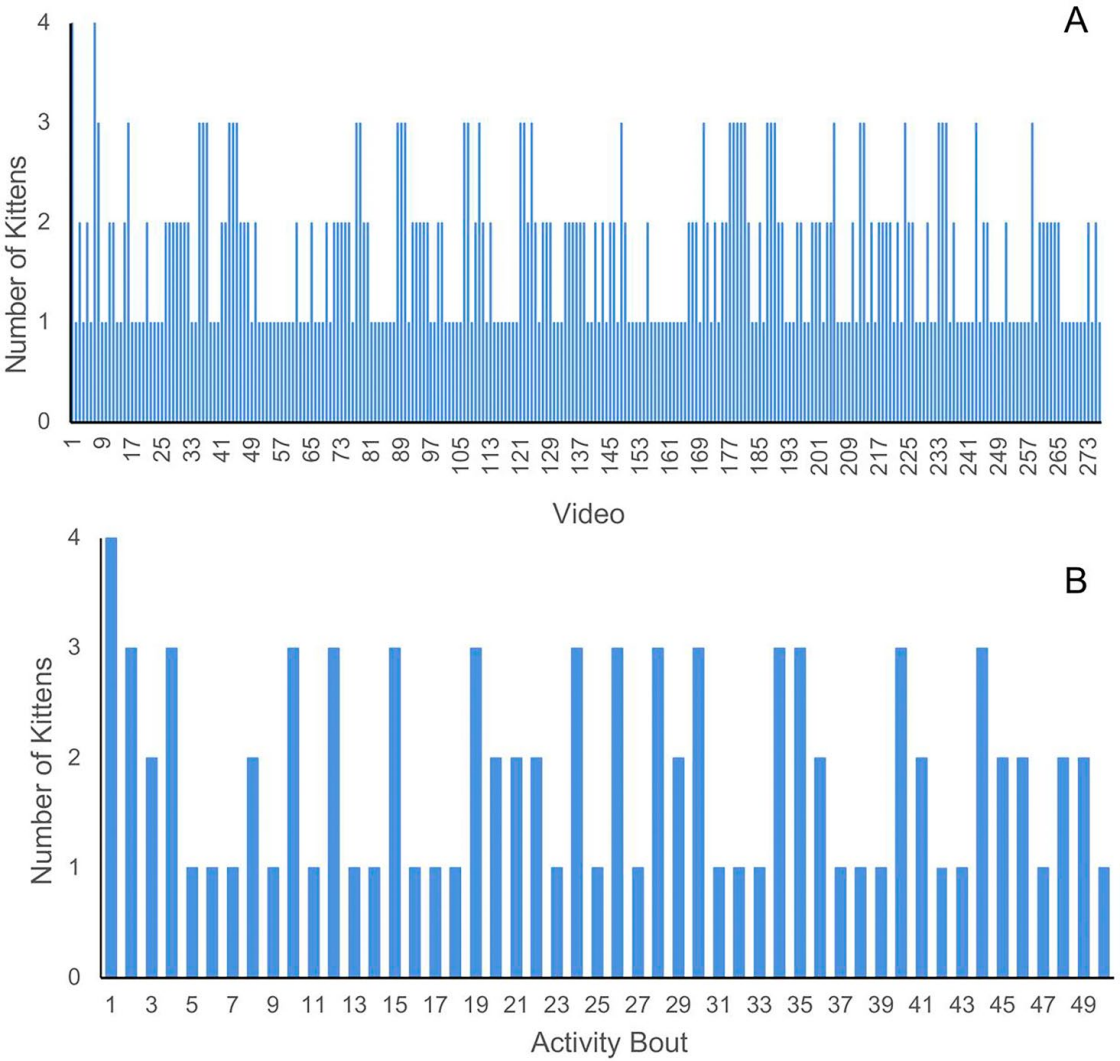
The count of number of kittens in each (A) video, and (B) activity bout documented at the nursery over the course of monitoring.

We confirmed the presence of four kittens, but this count of four was only observed on the first day of recording (Figure 3)—specifically in two of the seven videos that made up the initial activity bout (representing 2.6% of all activity bouts and just 0.5% of total videos). Following this initial activity bout, the maximum number of kittens we observed was three (observed in 28.0% of bouts and 13.8% of videos). Most frequently, either one (46.0% of activity bouts and 50.7% of videos) or two kittens (24.0% of bouts and 34.8% of videos) were recorded.

**FIGURE 4:**
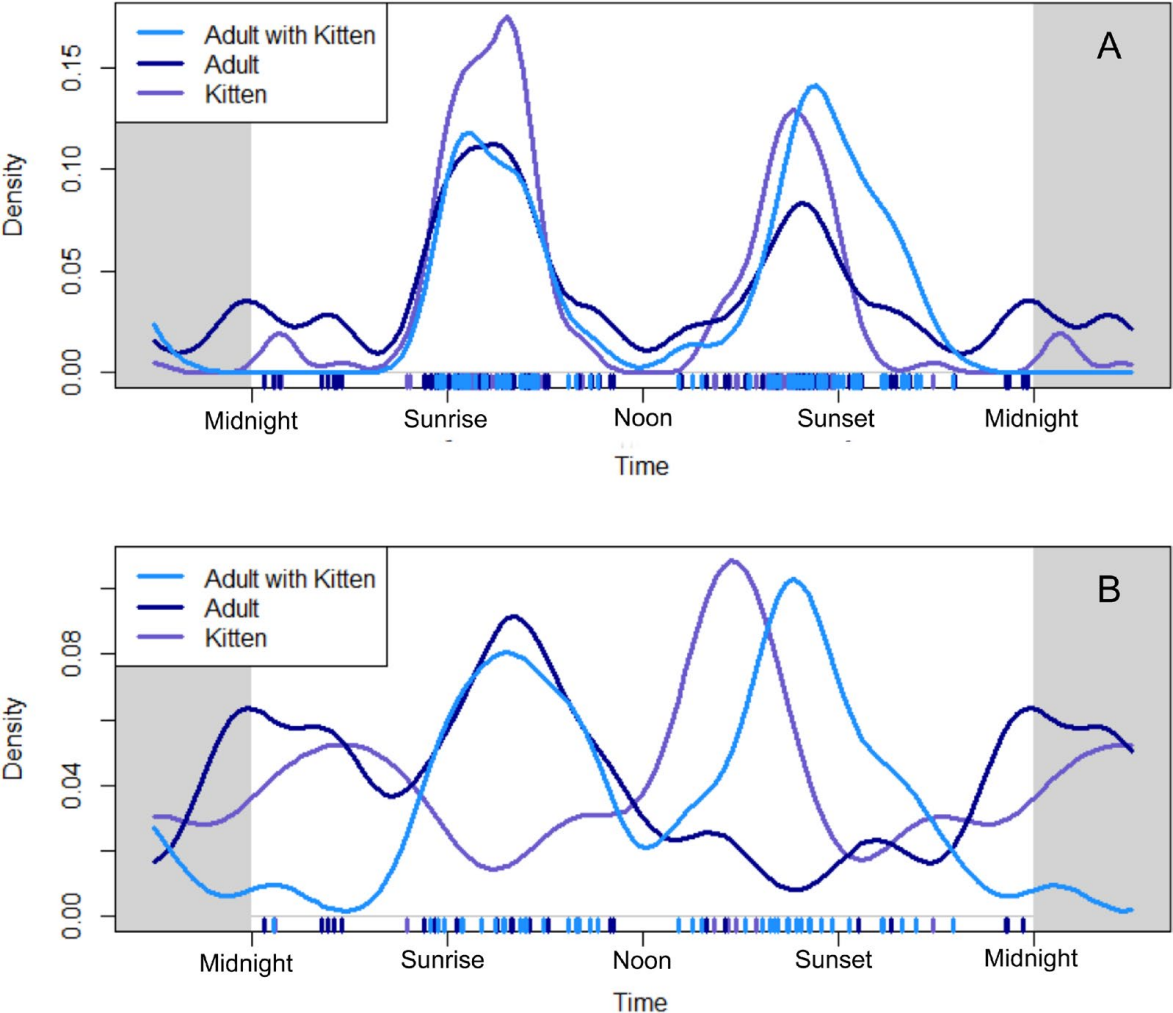
The diel activity (24 h) patterns of mountain lion classes (only the mother, only kittens, mother and kittens) documented at the nursery using data from each (A) video, and (B) activity bout over the course of monitoring.

Overall, mountain lions at the nursery exhibited a crepuscular pattern in diel activity, but this varied slightly among the mountain lion mother and kittens and more so depending on the temporal resolution (use of all videos or just events) of the data we analyzed. When we analyzed all of the videos, both kittens and the mother exhibited a crepuscular pattern, with a more pronounced peak around sunrise (Figure 4a). When we analyzed the start time of activity bouts, the mother was primarily active around sunrise, and kittens were crepuscular, with a peak in the late afternoon (Figure 4b). When the mother and kittens were both present, they had a crepuscular pattern with a slight peak around sunset for both data types.

Playing was the behavior most frequently observed at the nursery, occurring in 22 activity bouts (74 videos). Play typically consisted of kittens tree climbing or chasing and/or pouncing and wrestling each other (Video 1), but at times the mother would take part (Video 2). Across all recordings, vocalizations were detected in 42 videos (occurring within 20 distinct activity bouts). These vocalizations were either low growling purrs by the mother (Videos 1 and 2) or high‐pitched contact calls by a kitten (Video 3). We documented grooming in four videos (in four distinct bouts of activity); one case of allogrooming (the mother grooming a kitten; Video 3), and three cases of autogrooming (once by the mother and twice by kittens). The mother was observed carrying kittens in her mouth on two occasions (Video 4), both on the first day of observations. Despite extended surveillance, we did not record direct evidence of feeding or nursing. However, during post‐study inspections, we found remains of both porcupine ( *Erethizon dorsatum* ) and deer (*Odocoileus* sp.) within the immediate vicinity of the nursery. The mother also lacked fur around her nipples (Video 5), indicating ongoing nursing.

**VIDEO 3:** A mountain lion mother allogrooming a kitten, with vocalizations by the kitten. Video content can be viewed at https://onlinelibrary.wiley.com/doi/10.1002/ece3.71687 Video content can be viewed at https://onlinelibrary.wiley.com/doi/10.1002/ece3.71687.

**VIDEO 4:** A mother mountain lion carrying a kitten in her mouth, possibly as part of relocating to this nursery site. Video content can be viewed at https://onlinelibrary.wiley.com/doi/10.1002/ece3.71687 Video content can be viewed at https://onlinelibrary.wiley.com/doi/10.1002/ece3.71687.

**VIDEO 5:** A video of the mountain lion mother showing hairless nipples that are indicative of nursing. Video content can be viewed at https://onlinelibrary.wiley.com/doi/10.1002/ece3.71687 Video content can be viewed at https://onlinelibrary.wiley.com/doi/10.1002/ece3.71687.

## 4 Discussion

We had the unique opportunity to record observations from intensive, continuous monitoring of a mountain lion nursery for 7 days using a non‐invasive video camera trap. While most previous studies of mountain lion nurseries relied on telemetry data from tracking collars to understand broad patterns (e.g., Maehr et al. 1989; Benson et al. 2008; Jansen and Jenks 2012; Wilmers et al. 2013; Elbroch et al. 2015; Yovovich et al. 2020), our opportunistic study provided detailed natural history observations of neonatal mountain lions—a life stage rarely documented through direct observation. While our observations provide valuable insights into puma activity and behavior at a nursery, our relatively brief week of monitoring captured only a short snapshot of the denning period. Nonetheless, these observations demonstrate the value of camera traps for documenting rarely observed behaviors like denning—without disturbing these sensitive behaviors. Future studies should aim to monitor multiple nursery sites with longer temporal resolution and with multiple video camera traps positioned at various angles (without disturbing the nursery) to capture the full range of maternal care and rearing strategies, as well as kitten development and behaviors.

The fluctuating counts of kittens (and presence of the mother) observed throughout our monitoring of the nursery highlight a critical methodological challenge in studies using camera traps to estimate the occupancy and abundance of wildlife. Although we documented four kittens during the initial observation, subsequent recordings consistently captured only three or fewer individuals, with 85.5% of videos showing just one or two kittens at a time. We could not reliably distinguish individual kittens from the videos, limiting our ability to track individuals across time (or document individual behaviors). The absence of the fourth kitten beyond the first day could represent a mortality (considering that neonatal mountain lions are particularly vulnerable to mortality), but the variation in detection of individuals highlights the inherent limitations of camera traps to reliably detect all individuals within a specific area, and points to the potential for researchers to frequently underestimate actual counts. This underscores how detection probability varies substantially even within a setting where the number of individuals is known. Our findings suggest that multiple camera traps may be necessary to document species with complex three‐dimensional movement patterns, especially within dense vegetated habitats.

The patterns we observed in mountain lion diel activity at the nursery were generally crepuscular but varied based on the age class of the mountain lion and the temporal resolution of the data. The diel activity of mountain lions is often nocturnal or cathemeral (activity pattern of irregular intervals during the day or night) (Sweanor et al. 2008; Harmsen et al. 2011; Allen et al. 2024), but can shift by different classes of mountain lions—especially during sensitive behaviors (Allen et al. 2025). While mountain lions with offspring act as central place foragers (e.g., Yovovich et al. 2020), they are balancing kitten care with foraging—and the mother's heightened activity around sunrise may represent optimal hunting periods in this ecosystem. The divergent diel activity patterns we observed between the mother and kittens based on different temporal resolutions reveal important methodological considerations for camera trap studies about how different analytical approaches to the same dataset can yield substantially different ecological interpretations. Kittens tended to have fewer but longer bouts of activity (generating more videos per bout), while the mother had more frequent but shorter bouts of activity. This underscores the importance of clearly defining activity metrics and considering multiple analytical approaches, especially for species with complex social structures or age‐dependent activity patterns. This also raises the question of whether diel activity should be analyzed as the density of all activity (each photo/video), despite potential autocorrelation issues, or as a subset of data representing each bout of distinct activity for each individual. In some cases, using both video‐level and event‐level analyses can provide a more nuanced understanding of activity.

The behaviors we observed help our understanding of neonatal mountain lion behaviors at nurseries and their importance for social cohesion within the family group. The high frequency of play behavior observed among mountain lion kittens at the nursery suggests that social play (chasing, pouncing, wrestling) is a critical component of early development and may serve important roles in honing motor skills, practicing predatory behaviors, and establishing social bonds—as has been seen in other felids (Caro 1980). Occasional participation by the mother in play bouts underscores the social nature of these interactions and may reflect her role in stimulating or moderating kitten behavior. The frequent vocalizations we documented highlight the importance of acoustic communication in maintaining mother‐offspring contact around the nursery. The documentation of both allogrooming and autogrooming points to basic self‐maintenance and social bonding behaviors at the nursery. The presence of porcupine and deer remains near the nursery site provides evidence that the female was successfully hunting in the area and may have chosen this nursery location due to high prey resource availability. The absence of observed nursing behavior could indicate that nursing occurred primarily within the nursery and was not visible to our camera trap or that young were primarily feeding on the prey brought to the site by their mother. The two observations of the mother carrying kittens suggest young kittens that are likely still nursing, as this behavior is often associated with nursery relocation or repositioning neonatal young within the site.

The nursery we observed was located in the dense vegetation of a relatively remote area, both characteristics typical of mountain lion nurseries from other studies (Maehr et al. 1989; Benson et al. 2008; Elbroch et al. 2015). The dense structure likely provided multiple benefits, including physical cover from weather and visual concealment from potential threats, and highlights why these characteristics are often selected for as mountain lion nurseries (Maehr et al. 1989; Benson et al. 2008; Elbroch et al. 2015). The nursery being located within the protected and relatively undeveloped Gilbert‐Baker Wildlife Management Area further supports findings that female mountain lions preferentially choose areas with minimal human disturbance when rearing neonatal young in nurseries (Wilmers et al. 2013; Yovovich et al. 2020). These unique observations inform our understanding of rarely observed behaviors and activity of neonatal mountain lions, but our observation of a single nursery site limits generalizability across the species' range or even within the local, recently established Nebraska population. As mountain lions continue to reestablish populations in the Great Plains and other former parts of their range, maintaining access to such structurally complex habitats will likely be important for successful reproduction. These findings emphasize the need to identify and conserve potential nursery habitats, especially in landscapes experiencing increasing fragmentation or development (sensu Yovovich et al. 2020).

## Author Contributions

**Maximilian L. Allen:** conceptualization (lead), data curation (equal), formal analysis (equal), funding acquisition (equal), writing – original draft (lead). **Colin Croft:** conceptualization (supporting), data curation (equal), funding acquisition (equal), writing – review and editing (supporting). **Shannon P. Finnegan:** conceptualization (supporting), writing – review and editing (lead). **Bethany H. Warner:** conceptualization (supporting), data curation (equal), formal analysis (equal), writing – review and editing (supporting).

## Conflicts of Interest

The authors declare no conflicts of interest.

## Supporting information


**Data S1.** Supporting Information.

## Data Availability

The data for this manuscript is available as supporting information—S1.
